# Acute effects of decaffeinated coffee and the major coffee components chlorogenic acid and trigonelline on incretin hormones

**DOI:** 10.1186/1743-7075-8-10

**Published:** 2011-02-07

**Authors:** Margreet R Olthof, Aimée E van Dijk, Carolyn F Deacon, Robert J Heine, Rob M van Dam

**Affiliations:** 1 Department of Health Sciences and the EMGO Institute for Health and Care Research, VU University Amsterdam, the Netherlands; 2 Department of Biomedical Sciences, Panum Institute, University of Copenhagen, Denmark; 3 Diabetes Center, Department of Endocrinology, VU University Medical Center, Amsterdam, the Netherlands; 4 Department of Epidemiology and Public Health and Department of Medicine, Yong Loo Lin School of Medicine, National University of Singapore, Singapore

## Abstract

Coffee consumption is associated with a lower risk of type 2 diabetes. We tested the hypothesis that this is mediated by incretin hormones by measuring the acute effects of decaffeinated coffee and coffee components on GLP-1 and GIP concentrations. A randomized cross-over trial of the effects of 12 g decaffeinated coffee, 1 g chlorogenic acid, 500 mg trigonelline, and placebo on total and intact GLP-1 and GIP concentrations during an oral glucose tolerance test took place in fifteen overweight men. No treatment significantly affected the overall GLP-1 or GIP secretion pattern following an OGTT relative to placebo. Decaffeinated coffee slightly increased total GLP-1 concentration 30 minutes after ingestion (before the OGTT) relative to placebo (2.7 pmol/L, p = 0.03), but this change did not correspond with changes in glucose or insulin secretion. These findings do not support the hypothesis that coffee acutely improves glucose tolerance through effects on the secretion of incretin hormones. Chronic effects of coffee and its major components still need to be investigated.

## Findings

In prospective cohort studies, higher coffee consumption has been associated with a lower risk of type 2 diabetes [1,2]. Results were similar for caffeinated and decaffeinated coffee, suggesting that components other than caffeine are responsible for this association. Coffee is a major source of the phenolic compound chlorogenic acid [3] and the vitamin B3 precursor trigonelline [4] which have been shown to reduce blood glucose concentrations in animal studies [5-7]. One of the putative mechanisms underlying the beneficial effects of coffee on glucose homeostasis could include a stimulatory effect on the incretin hormones glucagon-like peptide 1 (GLP-1) and glucose-dependent insulinotropic peptide (GIP). Coffee components may affect GLP-1 secretion by delaying glucose absorption, resulting in a higher exposure of the distal ileum to glucose, and therefore stimulation of the intestinal K cells [8]. One study found that decaffeinated coffee increased GLP-1 concentrations [9] and two studies found that both decaffeinated coffee and caffeinated coffee intake decreased GIP concentrations [9,10]. These results suggest that coffee may exert a glucose-lowering response mediated by stimulating incretin hormones. Data on the effects of chlorogenic acid and trigonelline on incretin concentrations are lacking. We therefore investigated the acute effects of decaffeinated coffee and the coffee components chlorogenic acid and trigonelline on total and intact GLP-1 and GIP concentrations in humans.

Fifteen male, healthy, non-smoking, overweight (body mass index 25.0-35.0 kg/m^2^) coffee consumers were enrolled. All subjects provided written informed consent. The study design has been described previously [11]. Briefly, four supplements were tested in this cross-over trial: 12 grams of decaffeinated instant coffee granules (Nescafé Gold, Nestlé, The Netherlands); 1 g chlorogenic acid; 500 mg trigonelline; and 1 g mannitol as placebo. All supplements were dissolved in 270 mL water.

The study consisted of four visits separated by at least six days. During each visit participants ingested one of the supplements 30 minutes before a 75 g oral glucose tolerance test (OGTT). Seven venous blood samples were taken via a cannula in the antecubital vein on each visit following an overnight fast. The first two blood samples were taken 30 minutes and immediately before the start of the OGTT. The other 5 blood samples were taken 15, 30, 60, 90, and 120 minutes thereafter. For measurement of intact GLP-1 the DPP-4 inhibitor diprotin A (0.1 mmol/l, final concentration) was added immediately after collection.

Plasma concentrations of total GLP-1, intact GLP-1 and total GIP were determined as previously described [12,13]. Intra- and inter assay coefficients of variation were 2% and 5%, respectively for intact GLP-1 and <6% and <15%, respectively for both total GLP-1 and GIP. Results for glucose and insulin concentrations have previously been presented [11].

The area under the curve (AUC) values for GLP-1 and GIP were calculated using the trapezoidal method. Main treatment effects relative to placebo for AUC values and mean GLP-1 and GIP concentrations for individual time points were analyzed using linear mixed regression models. Bonferroni correction was used to adjust for multiple comparisons. All tests were two-sided and P values <0.05 were considered statistically significant. Analyses were conducted using SPSS 17.0.

**Figure 1:**
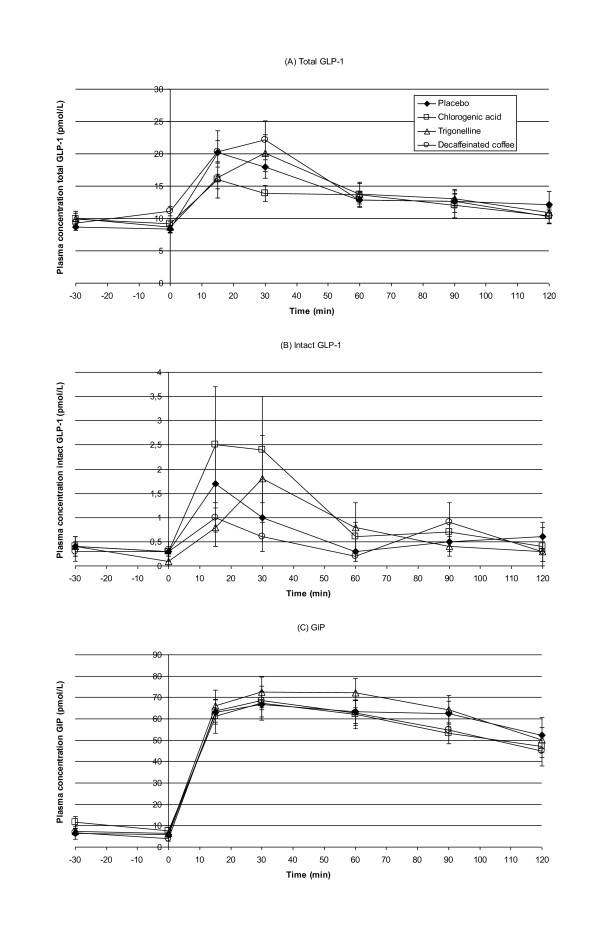
**Mean (± SE) plasma concentrations of total GLP-1 (A), intact GLP-1 (B) and GIP (C) before and throughout an OGTT, following ingestion of chlorogenic acid, decaffeinated coffee, trigonelline and placebo in 15 healthy overweight men**. Figure (A): At t = 0 decaffeinated coffee increased total GLP-1 concentration relative to placebo (p = 0.03).

None of the treatments affected the area under the curve values for total GLP-1, intact GLP-1, or total GIP during the OGTT (Table 1). Decaffeinated coffee slightly but significantly increased total GLP-1 concentration relative to placebo at 30 minutes after ingestion (before the OGTT) (2.7 pmol/L, 95%CI: 0.2;5.3, p = 0.03). Chlorogenic acid and trigonelline did not significantly affect incretin concentrations relative to placebo at any of the time points (Figure 1).

**Table 1:** Area under the curve during an oral glucose tolerance test (mean ± SE) following ingestion of chlorogenic acid, decaffeinated coffee, trigonelline and placebo in 15 healthy overweight men.

	TOTAL GLP-1	INTACT GLP-1*	GIP
		(pmol/L × 120 min)	
Placebo	1720 ± 180	83 ± 33	6984 ± 566**
Chlorogenic acid	1549 ± 178	140 ± 47	6656 ± 620
Decaffeinated coffee	1809 ± 152	66 ± 21	6725 ± 516
Trigonelline	1730 ± 160	94 ± 39	7512 ± 619

*Intact GLP-1 concentrations were very low and a substantial number was at the limit of detection and were reported as zero.

**n = 14 (1 missing value at time point 15 min.)

In this randomized cross-over trial in healthy overweight men, decaffeinated coffee, chlorogenic acid and trigonelline did not significantly affect the overall response of GLP-1 or GIP to an OGTT relative to placebo. This is in line with the finding that these supplements did not affect glucose and insulin area under the curves in this study [11]. Decaffeinated coffee slightly increased the total GLP-1 concentrations at 30 min after ingestion (before the OGTT) relative to placebo. This small change, although statistically significant, did not coincide with changes in glucose and insulin levels [11]. Therefore this is probably a chance finding.

The lack of an effect of decaffeinated coffee on GLP-1 in our study is not in line with results of Johnston et al. [9] who found an increased response. They also found that ingestion of caffeinated and decaffeinated coffee lowered GIP concentrations. This may be explained by the fact that Johnston et al. [9] gave 25 g glucose together with coffee, whereas we gave 75 g glucose 30 min after ingestion of the supplement. Potential differences in the type of coffee used in both studies probably do not explain the different results since the type of coffee and the concentration of chlorogenic acid were similar in the two studies [9,11]. Greenberg et al. [10] did not find an overall effect of decaffeinated coffee on GIP concentrations, which is in line with our results. However, they did find that decaffeinated coffee lowered GIP concentrations at 60 minutes following ingestion of the coffee (before the OGTT) relative to placebo and caffeine. In that study supplements were ingested 60 min before the OGTT. We do not have data on the GIP concentrations 60 min following ingestion of the supplements alone, because the OGTT was administered at 30 min after ingestion of the supplements.

In conclusion, acute ingestion of decaffeinated coffee and its major components chlorogenic acid and trigonelline did not affect GLP-1 and GIP responses during the oral glucose tolerance test. These findings do not support the hypothesis that the putative beneficial effect of coffee on the development of type 2 diabetes can be explained by improved GLP-1 and GIP responses to meals. Nevertheless, it cannot be excluded that habitual coffee consumption in contrast to acute administration may have an effect on incretin secretion.

## Competing interests

MRO, AEvD, CFD, RJH and RMvD have no relevant conflict of interest to disclose. RJH is currently employed at Eli Lilly and Company, Indianapolis, IN.

## Authors' contributions

All authors read and approved the final manuscript. MO, RH, and RvD conceived the study and obtained funding. MO and AvD coordinated the data collection and CD the laboratory measurements. MO conducted the data analysis and wrote the first draft of the manuscript. AvD, CD, RH & RvD contributed to the interpretation of the data and the revision of the manuscript.
